# Safety and Efficacy of the *Bordetella bronchiseptica* Vaccine Combined with a Vegetable Oil Adjuvant and Multi-Omics Analysis of Its Potential Role in the Protective Response of Rabbits

**DOI:** 10.3390/pharmaceutics14071434

**Published:** 2022-07-08

**Authors:** Xuemei Cui, Xiangfei Xu, Pan Huang, Guolian Bao, Yan Liu

**Affiliations:** 1 Institute of Animal Husbandry and Veterinary Science, Zhejiang Academy of Agricultural Sciences, Hangzhou 310021, China; maycui@zju.edu.cn (X.C.); 2020608021037@stu.zafu.edu.cn (X.X.); panhuang@zjnu.edu.cn (P.H.); 2 College of Animal Science and Technology, College of Veterinary Medicine, Zhejiang Agriculture and Forestry University, Hangzhou 311300, China; 3 College of Chemistry and Life Science, Zhejiang Normal University, Jinhua 321004, China

**Keywords:** vegetable oil adjuvant, vitamin E, ginseng saponins, *Bordetella bronchiseptica*, multi-omics analysis

## Abstract

Infectious respiratory diseases caused by *Bordetella bronchiseptica* (*Bb*) are seriously endangering the development of the rabbit industry in China. Unfortunately, no licensed vaccines are available for this pathogen. The present study was designed to determine whether the inactivated *Bb* antigen formulated with vegetable oil adjuvant (named E515) which contains soybean oil, vitamin E, and ginseng saponins, functions as a safe and effective vaccine (E515-*Bb*) against *Bb* infection in rabbits. Based on local and systemic reactions, both the E515 adjuvant alone and the E515-*Bb* vaccine exhibited good safety in rabbits. Immune response analysis implies that rabbits immunized with the E515-*Bb* vaccine produced significantly higher, earlier, and longer-lasting specific antibody responses and activated Th1/Th2/Th17 cell responses than those immunized with the aluminum hydroxide (Alum)-adjuvanted *Bb* vaccine (Alum-*Bb*) or *Bb* antigen alone. Moreover, the E515-*Bb* vaccine effectively protected rabbits from *Bb* infection. Additionally, integrated multi-omics analysis revealed that the immunoprotective effect of the E515-*Bb* vaccine was achieved through upregulation of the complement and coagulation cascades and cell adhesion molecule (CAM) pathways, and the downregulation of the P53 pathway. Overall, these results indicate that the E515-*Bb* vaccine is safe, elicits an efficient immune response and provides good protection against *Bb* infection in rabbits. Thus, the E515-adjuvanted *Bb* vaccine can be considered a promising candidate vaccine for preventing *Bb* infection.

## 1. Introduction

*Bordetella bronchiseptica* (*Bb*) causes respiratory infections in a variety of animal species, including swine, dogs, cats, rabbits, and occasionally humans [1]. Among these susceptible animals, rabbits are an economically important species. In recent years, rabbit farming has progressively become a unique livestock industry worldwide, especially in China [2]. According to FAOSTAT 2020 data sources (https://www.fao.org/faostat/en/#data/QCL), China is the largest rabbit meat producer and production region in the world. However, *Bb* poses a serious threat to the development of the rabbit industry, mainly because of its widespread distribution. It is reported that *Bb* is the important pathogen causing severe respiratory infection in rabbits in Fujian Province, which is one of the important rabbit-farming areas in China [3]. More research found that *Bb* can be isolated from approximately 80% of rabbits obtained from a breeding farm contaminated with *Bb* [4]. Moreover, *Bb* could pave the way for other pathogens, in turn leading to several secondary infections, resulting in significant economic losses for farmers [5,6]. Even worse, a case of *Bb* bacteremia in a patient with COVID-19 was observed [7], thereby demonstrating the potential of zoonotic transmission between animals and humans.

Improper antibiotic prevention and treatment further enhance the virulence of *Bb*, increasing the costs and difficulties of clinical treatment [3,8,9]. In contrast to antibiotics, antibacterial vaccines are regarded as powerful weapons [10]. Several *Bb* vaccines have been utilized routinely in dogs and pigs [11,12], whereas vaccines against *Bb* for use in rabbits have still not been developed, so it is urgent to develop an effective vaccine controlling the *Bb* infections in rabbits and preventing the potential rabbit–human transmission events. To date, *Bb* vaccine research mainly focuses on subunit vaccines or inactivated vaccines [6,13] in which mineral oil- or Alum-based adjuvants are common additive ingredients to enhance vaccine efficacy. Although the aforementioned vaccines could elicit antibodies against *Bb* in rabbits, safety problems associated with the components of the adjuvants lack consideration. Side effects, such as abscesses, cysts or necrosis, after the injection of mineral oil greatly limit its wide application. In addition, due to the poor metabolism of mineral oil in the animal body, it may remain in the meat that is intended for human consumption [14]. Our preliminary experiments also revealed that mineral oil as a *Bb* vaccine adjuvant resulted in severe side effects in rabbits (data not presented). Aluminum adjuvants are the most commonly used adjuvants in vaccines in the marker, but local reactogenicity and weak stimulation of cell-mediated immunity have been reported frequently [15,16]. For example, visible nodules, intense granulomatous, severe inflammatory response, as well as a loss of muscle fibers were observed in the injection site when the mice were vaccinated with an Alum containing a *Sporothrix schenckii* vaccine [17]. Since rabbits are farmed for not only meat but also fur, which is an important economic trait, selecting/developing adjuvants for rabbit vaccines should be performed in a careful manner.

E515 is a novel vegetable oil adjuvant that consists of soybean oil, vitamin E (VE), and ginseng saponins (GS). Compared to mineral oil and Alum, vegetable oil-derived E515 is much safer, which conforms to the standard of the Chinese Pharmacopoeia; VE is a nutritional factor, and GS is derived from a tonic herb [18,19]. Our previous studies verified that E515 significantly enhances the humoral and cellular immune responses induced by foot-and-mouth disease (FMD) vaccines in mice and Hu sheep [20,21]. On this basis, we further adjusted the adjuvant to make E515 more applicable for emulsification with whole-inactivated bacterial antigen. Compared with the subunit vaccine, the whole bacterial protein has a simpler preparation method and an inexpensive cost, which is more suitable for veterinary clinical application. Usually, a whole-inactivated bacteria vaccine has a good immunogenicity, and the incorporation of adjuvants can further enhance its immunogenicity. It was reported that ECMS-oil or Rg1-oil based adjuvant could significantly improve the immune effects of the whole-inactivated *Bb* vaccine [6,22]. Currently, people’s attention to adjuvants is mainly focused on improving the efficacy of vaccines, while less effort has been devoted to researching the safety of the adjuvants themselves.

This study was designed to evaluate the safety of E515 alone and the E515-adjuvanted *Bb* vaccine by observing local and systemic toxicities in rabbits. The adjuvant effect of E515 on immune responses induced by the *Bb* vaccine in rabbits was observed by investigating specific antibody responses, cellular responses, and long-lasting protective immunity. To further explore the potential of the E515-*Bb* vaccine to protect against *Bordetella bronchiseptica* infection, integrated multi-omics analysis of the transcriptome and proteome was performed, which provided guidance for the design of studies on animal vaccines. This study not only provides a solid foundation for safe and effective *Bb* vaccines, but also reveals new strategies for the application of adjuvants with traditional Chinese medicine and vitamins as the main components of animal vaccines. Thus, this study has theoretical significance and practical value.

## 2. Materials and Methods

### 2.1. Animals

Six-week-old New Zealand rabbits (Animal Center Co., Ltd., Zhejiang, China) were used for all experiments. Male and female rabbits were assigned randomly to each group. The animals were acclimatized for one week prior to use and kept in cages at a controlled temperature (20 ± 5 °C) and humidity (50% ± 10%) on a 12 h light–dark cycle and had free access to water. All animal experiments complied with the guidelines of the Animal Welfare and Use Committee of Zhejiang Academy of Agricultural Sciences (ZAAS, Hangzhou, China). The animal studies were approved by the ZAAS Ethics Committee (permit no. 2021ZAASLA17).

### 2.2. Vaccine Preparation

E515 adjuvant was made from soybean oil (Zhejiang Tian Yu Shan Medicinal Co., Ltd., Hangzhou, China), standardized GS (Hongjiu Ginseng Industry Co., Ltd., Baishan, China), and VE with a purity ≥ 96% (Sigma, Marlborough, MA, USA). Alum adjuvant was obtained from Thermo Scientific (Waltham, MA, USA). *Bb* (FX strain) was cultured as described in our previous report [23] and inactivated with 0.4% formalin. After an inactivation test, the bacteria were collected by centrifugation at 10,000 rpm for 10 min, then *Bb* bacteria were resuspended in the sterile PBS, after that, bacteria suspensions were concentrated to appropriate concentration. The whole-inactivated bacterial was adjuvanted with E515 or Alum at a ratio of 1:2 (*v*/*v*) or 1:1 (*v*/*v*), and the prepared vaccines contained inactivated 1.2 × 10^10^ CFU/mL *Bb* antigen. The E515-adjuvanted *Bb* vaccine was prepared as previously described [20,21] with some modifications, and each mL of the soybean oil contained both VE (100 µg) and GS (60 µg). Briefly, the mixture was emulsified at a shear rate of 16,000 rpm for 5 min (IKA T10 basic, ULTRA-TURRAX1, Staufen, Germany) to form an oil-in-water (O/W) emulsion. The protocol for mixing Alum with immunogen was performed according to the manufacturer’s instructions. Briefly, Alum was added dropwise to the *Bb* antigen with constant mixing, and then mixing was continued for 30 min after the adjuvant was added. To allow Alum to effectively adsorb antigen, it was freshly mixed with *Bb* antigen one day before immunization. The prepared vaccines were stored at 4 °C before use.

### 2.3. Experimental Design

**Experiment A:** To evaluate the safety of the adjuvant and vaccine. Rabbits (*n* = 9/group) were randomly assigned to six groups and were subcutaneously (s.c.) injected twice with 2 mL (2-fold higher than the dose in the immune experiment) of saline, inactivated *Bb* antigen (1.2 × 10^10^ CFU/mL), E515 adjuvant only, Alum adjuvant only, E515-*Bb* vaccine, or Alum-*Bb* vaccine. Each rabbit was inoculated on day 0 and boosted on day 14. All visible signs of local adjuvant-associated side effects ranged from redness and swelling to granulomas, sterile abscesses, lymphadenopathy, and skin ulceration, and any behavioral changes were recorded daily. Adjuvant-associated systemic reactogenicity symptoms, such as elevated temperature (on days 0–3, 14–17, and 21), decreased body weight (on days 0–3, 7, 14 and 21), and increased serum levels of acute phase proteins (APPs) and inflammatory factors (IL-1β and TNF-α) (on days 1, 3, 15, 17, and 21), were monitored at different time points (Figure S1A).

**Experiment B:** To investigate the immune response of the E515-*Bb* vaccine. Rabbits (*n* = 9/group) were s.c. injected twice with 1 mL of saline, inactivated *Bb* antigen (1.2 × 10^10^ CFU/mL), or *Bb* antigen adjuvanted with E515 or Alum at a 2-week interval. Two mL blood was drawn for serum collection. Serum samples were collected 7, 14, 21, and 28 days after boosting to measure the levels of *Bb*-specific IgG, soluble CD4 and CD8, Th1 cytokines (IFN-γ and IL-2), Th2 cytokines (IL-4 and IL-6), and Th17 cytokines (IL-17). Then, the rabbits were challenged by ear vein injection with live *Bb* (2.4 × 10^10^ CFU/rabbit), and the serum levels of IL-1β and TNF-α (1 day post-challenge) and endotoxin (1, 5, and 10 days post-challenge) were determined. The animal survival rate was monitored over the next 10 days. After that, spleen samples were collected from the rabbits (*n* = 3/group) for proteomic and transcriptomic analyses (Figure S1B).

**Experiment C:** To observe the long-term efficacy of the E515-*Bb* vaccine. Rabbits (*n* = 12/group) were s.c. injected with 1 mL of inactivated *Bb* antigen (1.6 × 10^10^ CFU/mL) or *Bb* antigen adjuvanted with E515 or Alum. Blood samples were collected 1, 2, 4, 6, and 8 months after immunization to measure *Bb*-specific IgG levels. Then, the rabbits were challenged by ear vein injection with live *Bb* (2.4 × 10^10^ CFU/rabbit). At 7 days post-challenge, the bacterial burdens in the lung and trachea were quantified, and the pathological lesions of lung tissues were evaluated by hematoxylin and eosin (HE) staining (Figure S1C).

### 2.4. Analysis of Bb-Specific Antibodies and Cytokine Levels

Briefly, the whole inactivated *Bb* protein fraction was added to a 96-well microtiter plate (2 μg/mL, 100 μL/well) and incubated at 4 °C overnight. Then, the plates were washed 3–5 times with phosphate-buffered saline containing 0.05% Tween-20 (PBST), and the wells were blocked with 5% skim milk and incubated at 37 °C for 2 h. After washing, 100 µL of the serum sample (1:100) was added to each well and incubated at 37 °C for 1 h. Then, 100 µL of goat anti-rabbit IgG (1:10,000) (Abcam, Cambridge, UK) was added and incubated at 37 °C for 1 h. After washing 3–5 times, tetramethyl-benzidine (TMB) reagent (100 μL/well) was added to the plates and incubated at 37 °C for 10–15 min in the dark. The reaction was terminated by adding 50 µL/well 2 M H_2_SO_4_, and the absorbance was measured by a microplate reader at 450 nm (Thermo-Multiskan FC, Shanghai, China).

The concentrations of IL-1β, TNF-α, IFN-γ, IL-2, IL-4, IL-6, IL-17, soluble CD4 and CD8, the APPs C-reactive protein (CRP) and serum amyloid P component (SAP), and endotoxin in the serum samples were determined by commercial ELISA kits (ML, Shanghai Enzyme Linked Biotech Co., Ltd., Shanghai, China) according to the manufacturers’ instructions.

### 2.5. Bacterial Loads and Histopathological Examination

The lungs and trachea were collected and homogenized by a Bio-Gen PRO-200 Homogenizer (Pro Scientific Inc., Oxford, MA, USA). Homogenates were serially diluted (1:10, 1:100, and 1:1000) in trypticase soy broth (TSB) (Oxoid, Basingstoke, UK), plated in duplicate on trypticase soy agar (TSA) (Oxoid, Basingstoke, UK) plates and incubated at 37 °C for 24 h. Colonies were counted for bacterial enumeration.

The lung tissues of infected rabbits from three different groups were removed under sterile conditions 7 days post-challenge. All lung samples were fixed with 4% paraformaldehyde (Solarbio, Beijing, China), embedded in paraffin wax, sliced, and stained with HE. Then, the slices were examined under a microscope for histological examination.

### 2.6. Transcriptomic Analysis

All live rabbits were sacrificed 10 days post-*Bb* challenge, and spleens of rabbits (*n* = 3/group) immunized with *Bb* antigen or *Bb* antigen adjuvanted with E515 or Alum were obtained for transcriptome sequencing. Total RNA was extracted with an RNeasy Mini Kit (Qiagen, MD, USA) according to the manufacturer’s instructions. The RNA concentration and integrity were measured by the Qubit 2.0 Fluorometer (Life Technologies, Carlsbad, CA, USA) and a Bioanalyzer 2100 system (Agilent Technologies, Santa Clara, CA, USA). Sequencing libraries were generated using a NEBNext Ultra^TM^ RNA Library Prep Kit for Illumina (NEB, Ipswich, MA, USA) in accordance with the manufacturer’s recommendations. Clustering of the index-coded samples was performed on a cBot Cluster Generation System using a TruSeq PE Cluster Kit v3-cBot-HS (Illumina, San Diego, CA, USA) according to the manufacturer’s instructions. After cluster generation, the library preparations were sequenced on an Illumina HiSeq 2500/X platform, and 125/150 bp paired-end reads were generated. Differential expression analysis of two conditions/groups was performed using the DESeq2 R package. DESeq2 provides statistical routines for determining differential expression in digital gene expression data using a model based on the negative binomial distribution. The resulting *p* values were adjusted by Benjamin and Hochberg’s method to determine the false discovery rate (FDR), and genes with a *p* < 0.05 and |fold change| ≥ 1.3 were deemed differentially expressed genes (DEGs). Additionally, all DEGs were subjected to Gene Ontology (GO) and Kyoto Encyclopedia of Genes and Genomes (KEGG) enrichment analyses. The sequencing data have been deposited into the NCBI database under the accession number PRJNA784157.

### 2.7. Proteomic Analysis

All live rabbits were sacrificed at 10 days post-*Bb* challenge, and spleens from rabbits (*n* = 3/group) immunized with *Bb* antigen or *Bb* antigen adjuvanted with E515 or Alum were collected for proteomic sequencing. First, protein was extracted from the rabbit spleen samples, and the protein concentration was determined with a bicinchoninic acid (BCA) kit (Beyotime, Shanghai, China). Proteins were precipitated using acetone, quantified with 4D label-free technique and analyzed using an LC–MS approach. Library preparation and sequencing were carried out by Jingjie Bio-Lab Co., Ltd. (Hangzhou, China). The separated peptides were analyzed on the Exploris 480^TM^ instrument (Thermo Scientific, Waltham, MA, USA), and the resulting MS/MS data were processed using the Proteome Discoverer search engine (v2.4.1.15). The FDR was adjusted to <1%, and the minimum score for modified peptides was set to >40. The minimum peptide length was set to 6. All the other parameters in Proteome Discoverer were set to the default values. Differentially expressed proteins (DEPs) were designated based on a *p* < 0.05 and |fold change| ≥ 1.5. All DEPs were subjected to GO and KEGG enrichment analyses. The sequencing data are available at ProteomeXchange under the identifier PXD029947.

### 2.8. Integrated Analysis of Transcriptome and Proteome Data

To explore the consistency between the transcriptomic and proteomic data, Pearson correlations among the different treatment groups were calculated. Based on the transcriptome and proteome screening criteria, DEGs and DEPs detected simultaneously were referred to as cor-DEGs-DEPs. Correlation analysis between the GO function and KEGG pathway information in the transcriptome and proteome was performed, and the similarities and differences between gene function and metabolic pathways were compared between each group pair.

### 2.9. Validation of DEGs/DEPs by Real Time-qPCR/PRM

A total of 12 DEGs and 13 DEPs were selected to verify the sequencing results by real-time quantitative PCR (RT–qPCR) and parallel reaction monitoring (PRM). All primers for DEGs were designed using NCBI Primer BLAST and synthesized by Sangon Biotech (Table S1). Quantitative PCR was performed using a TransScript^®^ II Green One-Step RT–qPCR SuperMix Kit (TRAN, Beijing, China) on an ABI Prism 7500 (Bio–Rad, Hercules, CA, USA) and analyzed by the comparative CT method [24] The PCR cycling conditions were set as follows: 50 °C for 5 min; 94 °C for 30 s; and 40 cycles of amplification at 94 °C for 5 s and 60 °C for 30 s (annealing and elongation). The PRM assay was performed as we previously described [23].

### 2.10. Statistical Analysis

GraphPad Prism 7.0 software (GraphPad Software, San Diego, CA, USA) was used for data analysis. Multiple comparisons were conducted by two-way ANOVA, followed by Tukey’s multiple comparisons test, or by one-way ANOVA, followed by Tukey’s multiple comparisons test. The results are expressed as the mean ± SE, and *p* < 0.05 was considered statistically significant.

## 3. Results

### 3.1. Safety Estimation of E515 and the E515-Adjuvanted Bb Vaccine

**Figure 1:**
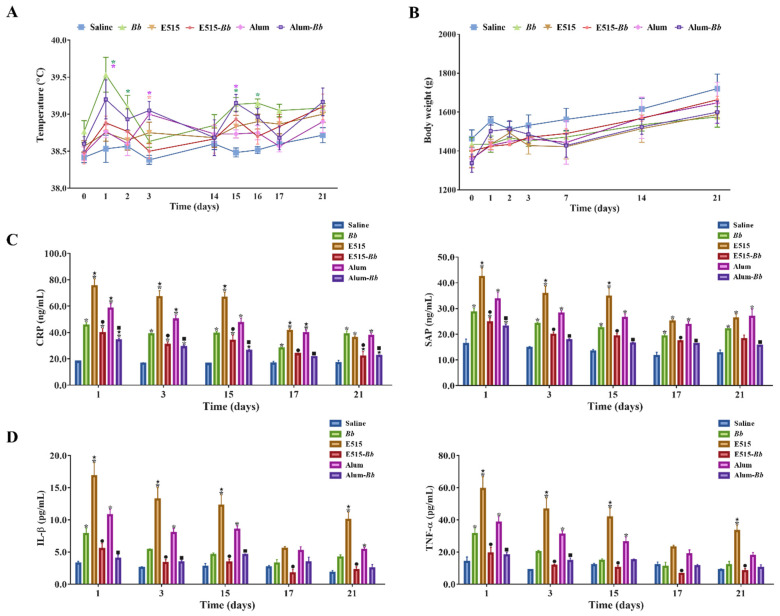
Safety estimation of the adjuvanted *Bb* vaccine. Rabbits (*n* = 9/group) were subcutaneously (s.c.) injected twice at a 2-week interval with 2 mL of saline, inactivated *Bb* antigen (1.2 × 10^10^ CFU/mL), E515 only, Alum only, or inactivated *Bb* antigen adjuvanted with E515 (E515-*Bb*) or Alum (Alum-*Bb*). (**A**,**B**) Temperature and weight changes at different time points after immunization (* *p* < 0.05 compared with the saline group, the colors of * are in line with the indicated groups). (**C**) I Serum levels of acute phase proteins (CRP and SAP). (**D**) Concentrations of serum cytokines (IL-1β and TNF-α). Data are presented as the mean ± SE. * *p* < 0.05 vs. Saline group, ^★^ *p* < 0.05 vs. *Bb* group, ^●^ *p* < 0.05 *Bb* vs. E515-*Bb* group, ^■^ *p* < 0.05 *Bb* vs. Alum-*Bb* group.

To assess the safety profiles of E515, adjuvant-associated local and systemic toxicities were evaluated in rabbits immunized with E515 alone or the E515-adjuvanted *Bb* vaccine. No obvious local erythema, swelling or lesions at the injection site post-vaccination were observed in any of the rabbits. The systemic reactogenicity results are shown in Figure 1. The temperature and weights of rabbits in the E515 and E515-adjuvanted *Bb* vaccine groups did not significantly differ from those in the saline group (control group) (Figure 1A,B); although, rabbits in the *Bb* antigen, Alum, and Alum-adjuvanted *Bb* vaccine groups had higher temperatures 1–3 days post-vaccination than those in the control group. However, the temperatures were still in the normal range (>40 °C was considered a fever) (Figure 1A). Moreover, the concentrations of the APPs and the proinflammatory cytokines IL-1β and TNF-α were significantly increased in rabbits injected with E515 adjuvant only compared to those in the E515-*Bb* vaccine group, whereas no significant differences were observed between the E515-*Bb* vaccine and control groups on days 17 and 21. The same results were also found for Alum (Figure 1C,D).

### 3.2. Evaluation of the Immune Effect of the E515-Bb Vaccine

**Figure 2:**
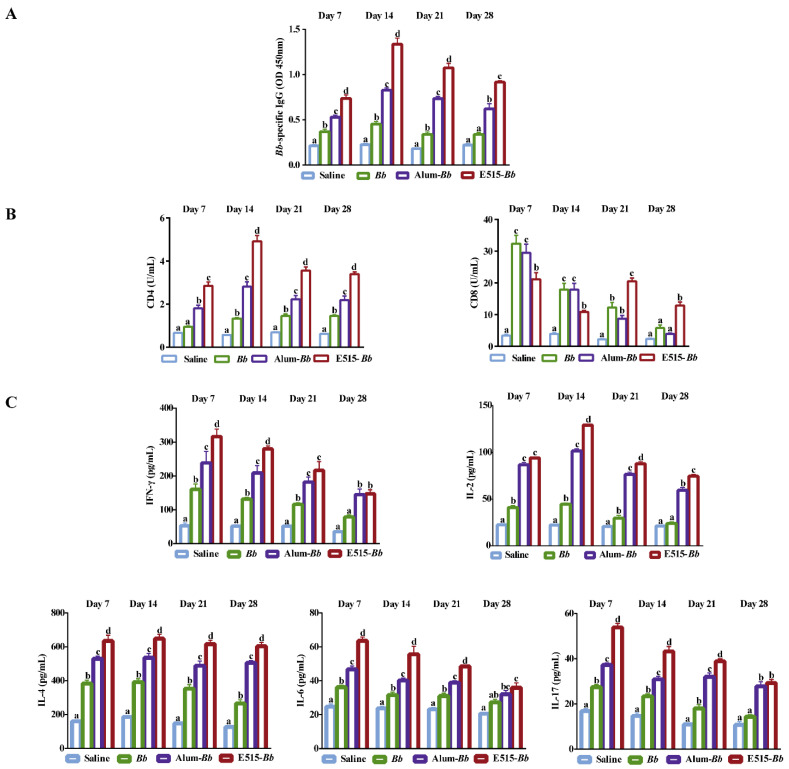
Immune effects of the E515-*Bb* vaccine. Rabbits (*n* = 9/group) were s.c. injected twice at a 2-week interval with 1 mL of saline, inactivated *Bb* antigen (1.2 × 10^10^ CFU/mL), or inactivated *Bb* antigen adjuvanted with E515 or Alum. Serum samples were collected 7, 14, 21, and 28 days after boosting to measure *Bb*-specific IgG (**A**), soluble CD4 and CD8 (**B**), Th1 cytokine (IFN-γ and IL-2), Th2 cytokine (IL-4 and IL-6) and Th17 cytokine (IL17) levels (**C**). Data are presented as the mean ± SE. Bars labeled with different lowercase letters (e.g., “a” and “b”) are significantly different (*p* < 0.05), and bars labeled with same or common lowercase letters are not statistically different (e.g., “a” and “ab”), (*p* ≥ 0.05).

To evaluate the immune effects of the E515-*Bb* vaccine on humoral and cellular immune responses, rabbits were immunized with saline, *Bb* antigen or *Bb* antigen adjuvanted with E515 or Alum, and blood samples were collected to detect *Bb*-specific antibodies, soluble CD4 and CD8, and serum cytokine levels. The levels of *Bb*-specific IgG were higher in the E515-*Bb* and Alum-*Bb* vaccine groups than in the *Bb* antigen group. However, the E515-*Bb* vaccine elicited significantly higher production of the IgG antibody than Alum-*Bb* from 7 to 28 days after the booster injection (Figure 2A). The data in Figure 2B shows that the soluble CD4 levels were significantly increased in E515-*Bb* vaccine-immunized rabbits compared to those in other groups at different time points post-boost, while the soluble CD8 levels were obviously higher in the E515-*Bb* vaccine group than in the other groups in the latter two weeks. The cytokine results showed significantly higher IFN-γ, IL-2, IL-4, IL-6, and IL-17 levels in rabbits immunized with the two adjuvanted *Bb* vaccine groups; whereas, the production of IL-6 did not significantly differ between the Alum-*Bb* and *Bb* antigen groups on day 28 (Figure 2C). Compared with those in the Alum-*Bb* vaccine group, the rabbits in the E515-*Bb* vaccine group secreted higher levels of IFN-γ (except on days 21 and 28), IL-2 (except on day 7), IL-4, IL-6, and IL-17 (except on day 28).

### 3.3. Assessment of the Role of the E515-Bb Vaccine in Protecting Rabbits from the Bb Challenge

**Figure 3:**
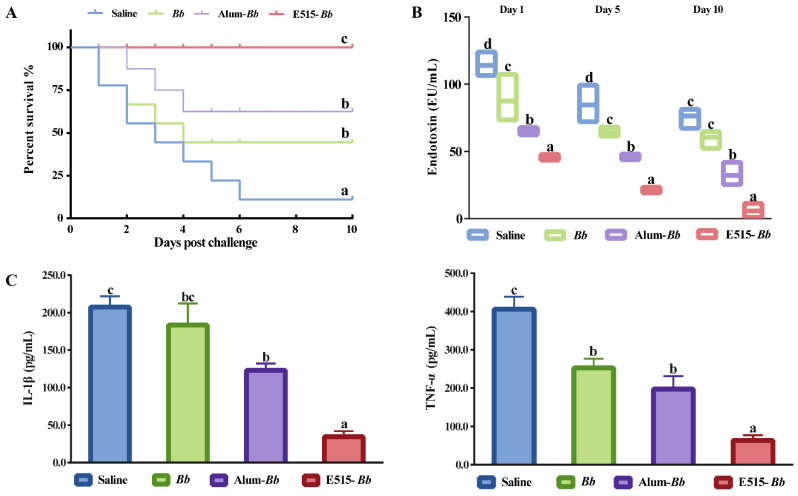
Protective effect of the E515-*Bb* vaccine post *Bb* challenge. Rabbits (*n* = 9/group) were challenged 4 weeks after boosting by ear vein injection with live *Bb* (2.4 × 10^10^ CFU/rabbit). The animal survival rate was monitored over the next 10 days (**A**), the serum level of endotoxin was detected 1, 5, and 10 days after infection (**B**), and the serum levels of IL-1β and TNF-α were determined 24 h after infection (**C**). Data are presented as the mean ± SE. Bars labeled with different lowercase letters (e.g., “a” and “b”) are significantly different (*p* < 0.05), and bars labeled with same or common lowercase letters are not statistically different (e.g., “a” and “ab”), (*p* ≥ 0.05).

To investigate the immunoprotective effect of the E515-*Bb* vaccine, the survival rate and serum endotoxin and proinflammatory cytokine levels were observed 10 days post-challenge. As presented in Figure 3A, rabbits immunized with the Alum-*Bb* vaccine or *Bb* antigen were better protected (62.5% and 44.4% survival) than those in the saline group (11% survival), while the best survival rate was found in the E515-*Bb* group (100% survival) after challenge. Compared with those in the saline group, the concentrations of endotoxin and the proinflammatory cytokines IL-1β and TNF-α were significantly lower in the two adjuvant vaccine groups, and the lowest levels of these indicators were observed in the E515-*Bb* vaccine group (Figure 3B,C).

### 3.4. Evaluation of the Long-Term Protection Provided by the E515-Bb Vaccine

**Figure 4:**
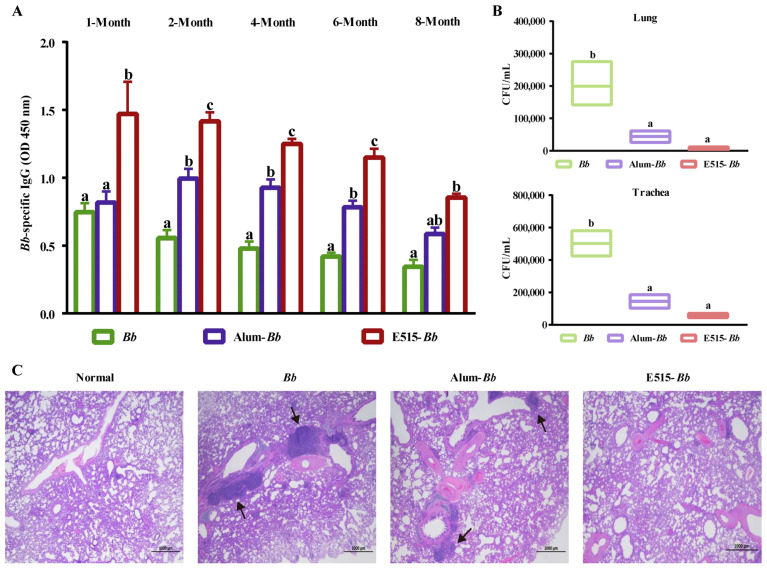
Evaluation of the long-term protection produced by the E515-*Bb* vaccine. Rabbits (*n* = 12/group) were s.c. injected with 1 mL of inactivated *Bb* antigen (1.6 × 10^10^ CFU/mL) or inactivated *Bb* antigen adjuvanted with E515 or Alum. Serum samples were collected 1, 2, 4, 6, and 8 months after immunization to measure *Bb*-specific IgG levels (**A**). Then, rabbits were challenged by ear vein injection with live *Bb* (2.4 × 10^10^ CFU/rabbit). Seven days after infection, bacterial burdens in the lung and trachea were quantified (*n* = 5) (**B**), and HE staining of lungs from infected rabbits was performed, arrows show area of inflammatory cell infiltration (**C**). Data are presented as the mean ± SE. Bars labeled with different lowercase letters (e.g., “a” and “b”) are significantly different (*p* < 0.05), and bars labeled with same or common lowercase letters are not statistically different (e.g., “a” and “ab”), (*p* ≥ 0.05).

To investigate the long-lasting protective ability of the E515-adjuvanted vaccine, rabbits were immunized with *Bb* antigen or *Bb* antigen adjuvanted with E515 or Alum. *Bb*-specific antibodies were detected at 1, 2, 4, 6, and 8 months after immunization, and the bacterial loads in the lung and trachea, as well as histological changes in the rabbit lungs, were observed 7 days post-*Bb* challenge. We found that the E515-adjuvanted *Bb* vaccine elicited significantly higher levels of antibody at 1, 2, 4, and 6 months after immunization than the other two groups, while the serum IgG levels did not significantly differ between the two adjuvant groups at 8 months (Figure 4A). Additionally, rabbits in the E515-*Bb* group exhibited significantly reduced *Bb* burdens in their lungs and tracheas compared with those in the *Bb* antigen group. No significant difference was observed between the E515 with Alum (Figure 4B). The histological changes at 7 days post-challenge revealed that the lungs in the E515-*Bb* group had a normal structure and clearer alveoli, whereas rabbits in the *Bb* antigen and Alum groups exhibited extensive lung lesions, inflammatory cell infiltration and structural damage (Figure 4C).

### 3.5. Differential Gene Expression Analysis

**Figure 5:**
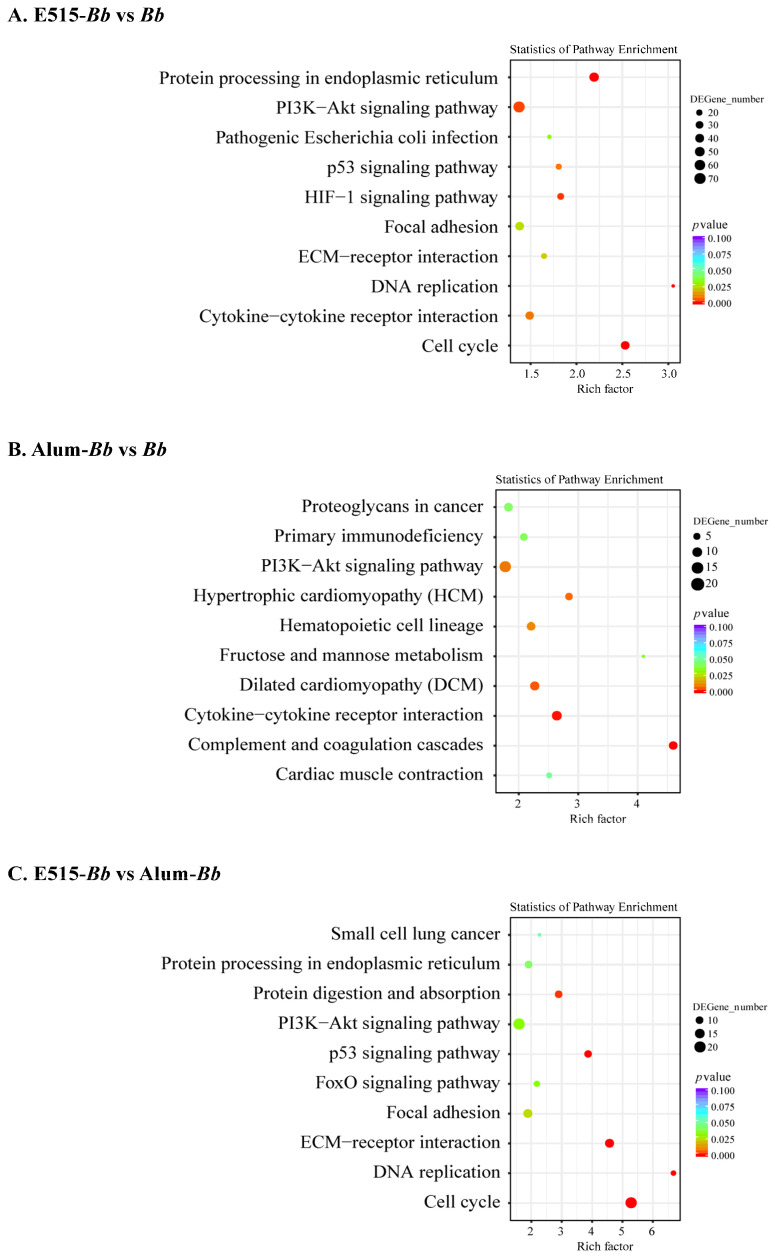
Kyoto Encyclopedia of Genes and Genomes (KEGG) pathway enrichment analysis of DEGs in two comparisons. (**A**) E515-*Bb* vs. *Bb*, (**B**) Alum-*Bb* vs. *Bb*, and (**C**) E515-*Bb* vs. Alum-*Bb*. The X-axis represents the value of the rich factor. The Y-axis represents the enrichment term of the pathway. The size of the dot represents the number of DEGs. The color represents *p* value.

To illustrate the immune-protective effects of the E515-*Bb* vaccine against *Bb* challenge, spleens from the infected rabbits were collected for RNA-sequencing (RNA-seq) analysis. There were 1905 DEGs (869 up- and 1036 downregulated) in E515-*Bb* vs. *Bb*, 427 DEGs (209 up- and 218 downregulated) in Alum-*Bb* vs. *Bb*, and 457 DEGs (147 up- and 310 downregulated) in E515-*Bb* vs. Alum-*Bb* (Figure S2). A hierarchical heatmap showed obviously distinct gene expression levels in every two-group comparison, and the DEGs in biological replicates clustered together, which exhibited good repeatability within the group (Figure S3). The functional enrichment of DEGs was performed by GO enrichment analysis. The 5–10 most activated immune-related GO terms are listed for every two-group comparison (Table S2). GO terms that were identified in the E515-*Bb* group were significantly enriched in cell division, animal organ regeneration, positive regulation of T cell differentiation and cellular response to cytokines (TGF, IL-4) when compared with the *Bb* or Alum-*Bb* group. Pathways involved in the cell cycle, DNA replication, protein processing in the endoplasmic reticulum, p53 signaling pathway and protein digestion and absorption showed higher levels of enrichment in the E515 adjuvant group than in the other two groups (Figure 5A,C), and all these pathways were downregulated. The complement and coagulation cascades and cytokine−cytokine receptor interaction pathways were obviously activated by the Alum adjuvant compared to the *Bb* antigen, among which the former was upregulated, and the latter was downregulated (Figure 5B).

### 3.6. Differential Protein Expression Analysis

**Figure 6:**
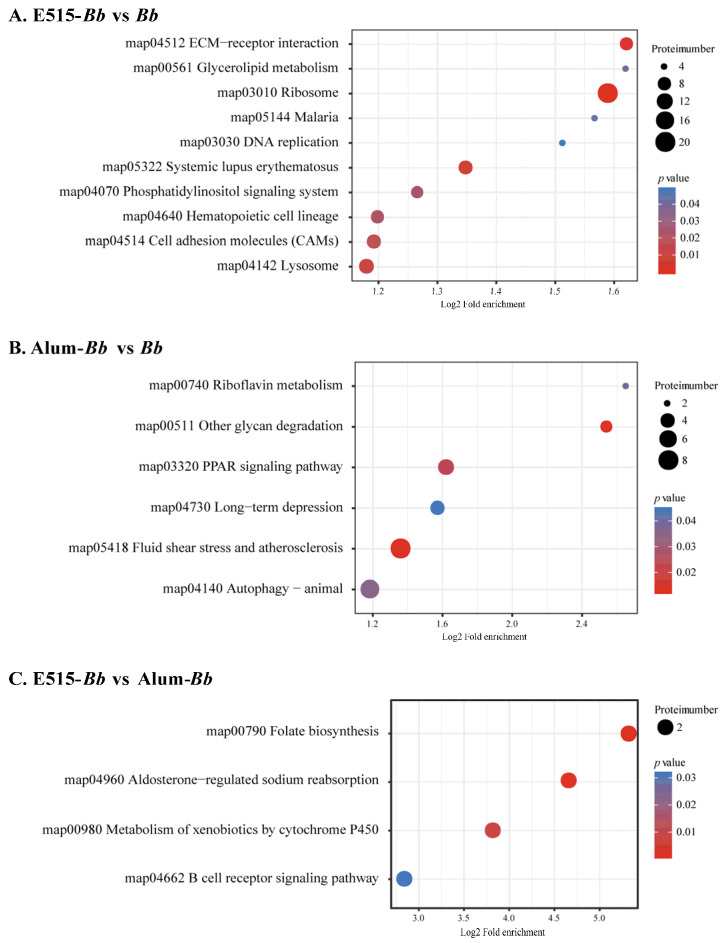
KEGG pathway enrichment analysis of DEPs in two comparisons. (**A**) E515-*Bb* vs. *Bb*, (**B**) Alum-*Bb* vs. *Bb*, and (**C**) E515-*Bb* vs. Alum-*Bb*. The X-axis represents the value of Log2 fold enrichment. The Y-axis represents the enrichment term of pathway. The size of the dot represents the number of DEPs. The color represents *p* value.

In this study, a 4D label-free proteomic approach was used to detect DEP profiles in rabbit spleens. Protein identification and quantitation showed that 5195 proteins were identified based on matches to 50,161 unique peptides (Figure S4). Box plots were generated to visualize the repeatability within each group (Figure S5). Compared with the *Bb* antigen group, 307 DEPs (138 up- and 169 downregulated) and 249 DEPs (94 up- and 155 downregulated) were identified in the E515-*Bb* and Alum-*Bb* groups, respectively. A total of 63 DEPs (27 up- and 36 downregulated) were screened between the E515-*Bb* and Alum-*Bb* groups (Figure S6). GO enrichment results showed that the terms regulation of leukocyte and lymphocyte activation, activation of immune response and apoptotic cell clearance were significantly activated in the E515-*Bb* group compared to the *Bb* group. GO terms related to antioxidant activity, cellular oxidant detoxification or detoxification, and negative regulation of leukocyte proliferation and activation were enriched in Alum-*Bb* compared with *Bb*. In addition, the ion channel binding- and ion channel regulator activity-related GO terms were significantly and differentially abundant between the two adjuvant groups (Table S3). KEGG enrichment results revealed that, compared to the *Bb* group, ribosome and ECM−receptor interactions were the most significantly and differentially abundant immune-related pathways activated by the E515 adjuvant, and the PPAR signaling pathway was obviously enriched in the Alum-treated group (Figure 6A,B). Moreover, the B-cell receptor signaling pathway was found to be significantly upregulated in E515-*Bb* vs. Alum-*Bb* (Figure 6C).

### 3.7. Correlation Analysis of the Transcriptome and Proteome

**Figure 7:**
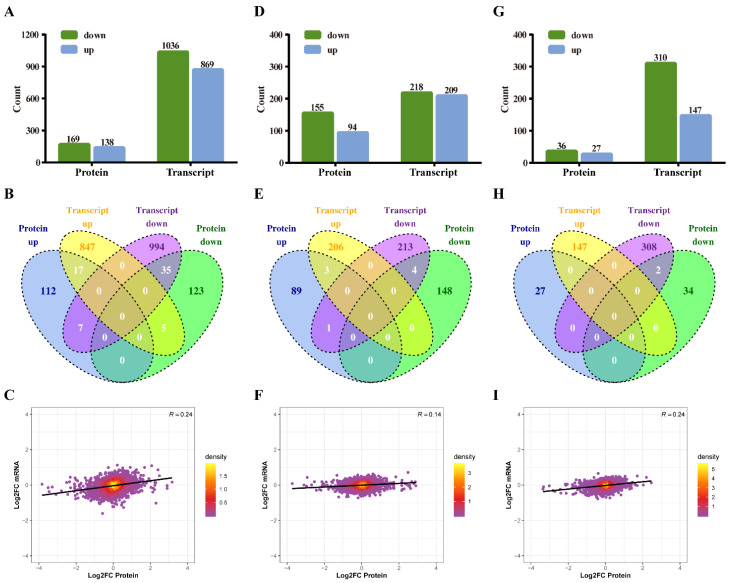
Correlation analysis of proteomics and transcriptomics. Rabbits (*n* = 9/group) were challenged 4 weeks after boosting by ear vein injection with live *Bb* (2.4 × 10^10^ CFU/rabbit). Spleens (*n* = 3/group) were harvested 7 days after infection and used for proteomic and transcriptomic analyses. (**A**,**D**,**G**) The numbers of differentially expressed proteins (DEPs) and genes (DEGs) quantified in the protein and transcript libraries. (**B**,**E**,**H**) Venn diagrams of up- and downregulated DEPs and DEGs. (**C**,**F**,**I**) Scatterplot of the relationship between genes identified in the protein and transcript libraries. (**A**–**C**) E515-*Bb* vs. *Bb*, (**D**–**F**) Alum-*Bb* vs. *Bb*, and (**G**–**I**) E515-*Bb* vs. Alum-*Bb*.

**Figure 8:**
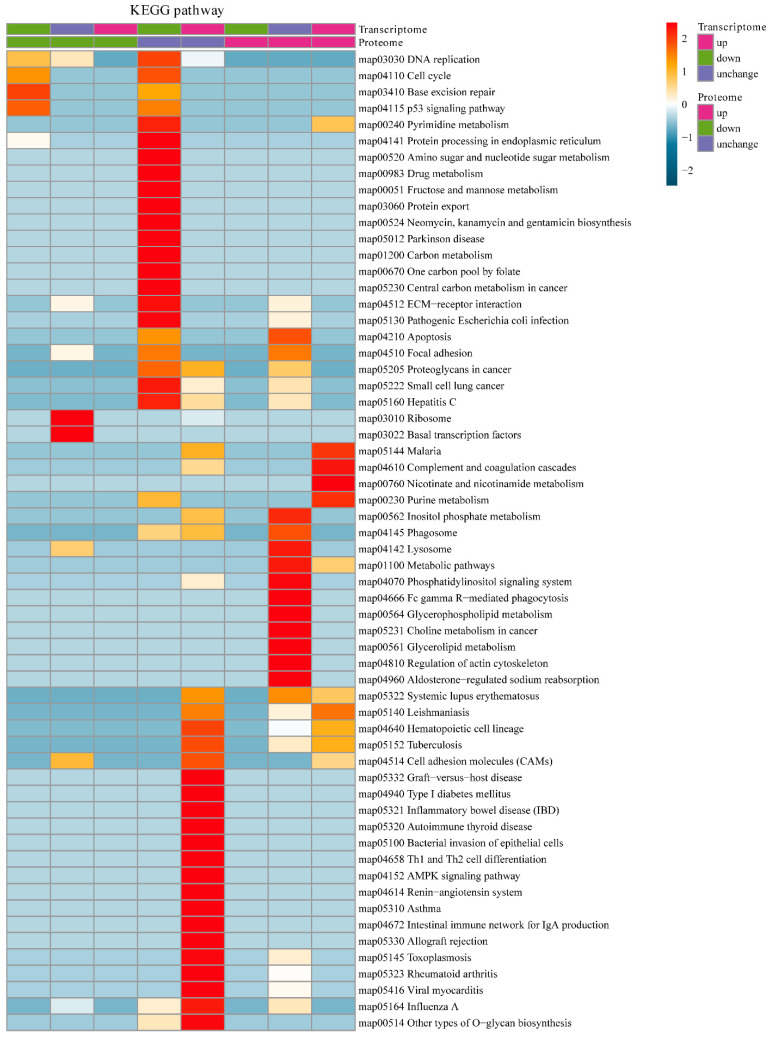
KEGG pathway enrichment-based clustering analysis of DEGs and DEPs in E515-*Bb* vs. *Bb*.

**Figure 9:**
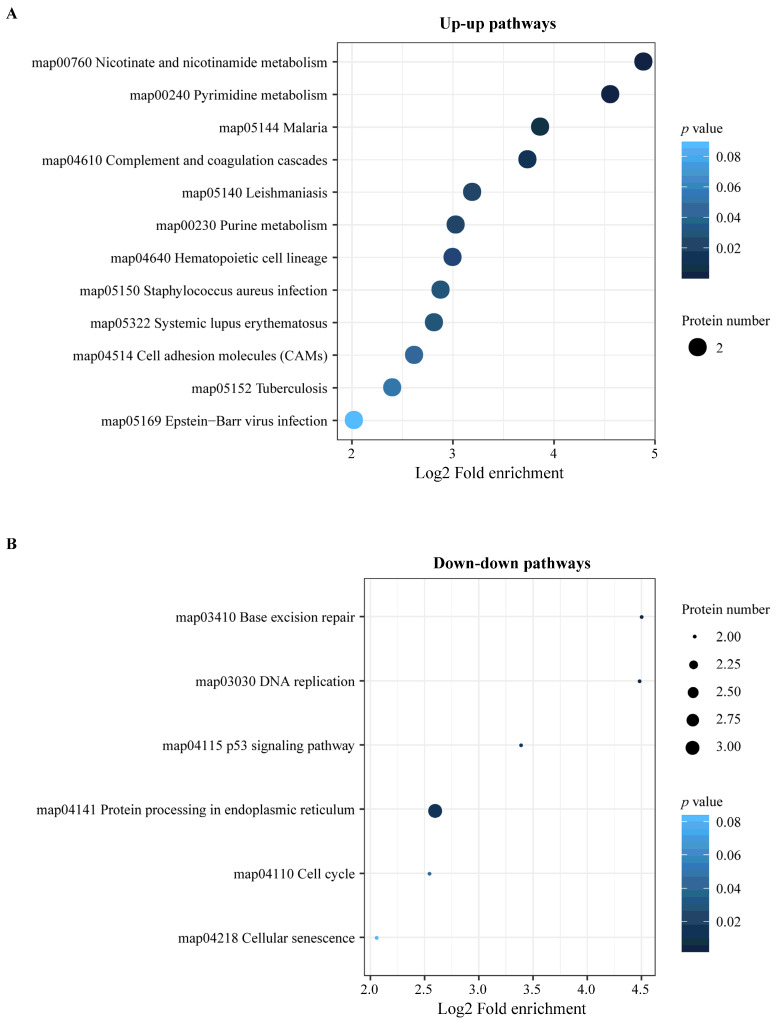
KEGG pathway enrichment analysis of cor-DEGs-DEPs in E515-*Bb* vs. *Bb*. (**A**) Up-up pathways. (**B**) Down-down pathways. The X-axis represents the value of Log2 fold enrichment. The Y-axis represents the enrichment term of pathway. The size of the dot represents the number of DEPs. The color represents *p* value.

The integration of the transcriptome and proteome analysis showed that in E515-*Bb* vs. *Bb*, 1905 DEGs (869 up- and 1036 downregulated) and 307 DEPs (138 up- and 169 downregulated) were identified (Figure 7A). Additionally, 64 cor-DEGs-DEPs were screened, among which 52 cor-DEGs-DEPs (17 up- and 35 downregulated) showed the same expression trend, whereas 12 showed the opposite trend (Figure 7B). The numbers of DEGs/DEPs and cor-DEGs-DEPs in Alum-*Bb* vs. *Bb* were 427, 249 and 8, in E515-*Bb* vs. Alum-*Bb* were 457, 63, and 2 (Figure 7D,E,G,H). The Pearson’s correlation coefficients between the transcriptome and proteome were 0.24, 0.14, and 0.24 in every two-group comparison, indicating a positive correlation (Figure 7C,F,I). Seven immune-related cor-pathways were found to be specifically enriched in E515-*Bb* vs. *Bb,* including 2 up-upregulated pathways (complement and coagulation cascades pathway and cell adhesion molecules, CAMs) and 5 down-downregulated pathways (base excision repair, DNA replication, p53 signaling pathway, protein processing in the endoplasmic reticulum and cell cycle) (Figure 8 and Figure 9). Meanwhile, no cor-pathways were found in Alum-*Bb* vs. *Bb* or E515-*Bb* vs. Alum-*Bb* (Figures S7 and S8). Detailed information for cor-pathways is shown in Table S4.

### 3.8. Validation of DEGs/DEPs by RT–qPCR/PRM

To verify the results of RNA-seq and 4D label-free LC–MS analysis, 12 DEGs and 13 DEP candidates were selected for validation by RT–qPCR and PRM. The trends in these results were similar to those in the RNA-seq and label-free LC–MS analyses (Figure S9), which suggested that the transcriptome and proteome data were credible.

## 4. Discussion

The rabbit industry is progressively becoming a highly specialized and unique livestock industry in China and worldwide because of the value of rabbit fur and meat. Currently, this industry is challenged by infectious diseases caused by *Bordetella bronchiseptica*, and even by the emergence of multidrug-resistant *Bb* due to high antibiotic use. Consequently, the search for safe and effective vaccines to prevent *Bb* infection are urgently needed. In this study, we described an inactivated *Bb* vaccine adjuvanted with a novel vegetable oil adjuvant E515 and evaluated the immunogenicity of the E515-*Bb* vaccine. An ideal adjuvant should maximize vaccine immunogenicity without compromising tolerability or safety concerns [25]. In addition to obvious local reactions, adjuvants are limited by their systemic reactogenicity, which induces local tissue damage in animals [26]. To conform to the veterinary vaccine quality standards, repeat-dose toxicity studies were conducted in accordance with the China veterinary pharmacopoeia. The results showed that both the E515 adjuvant alone and the E515-adjuvanted *Bb* vaccine had no local and systemic toxicities in rabbits (Figure 1A,B).

APPs refer to a group of proteins, such as CRP and SAP, that are synthesized primarily by hepatocytes [27,28]. Considering that various adverse events induced by vaccines are related to APPs [29,30,31], the well-known rabbit acute phase proteins CRP and SAP were quantified as a measure of vaccine safety [31]. The APP analysis results indicate that the concentrations of CRP and SAP were significantly increased in rabbits injected with E515 or the Alum adjuvant only compared to those in the two adjuvanted vaccine groups (Figure 1C). The same phenomenon is reported in rabbits injected with only the AS03 adjuvant [32], which might have been due to the structure of the E515-*Bb* vaccine, which is similar to that of the oil-in-water emulsion of AS03 [33]. The antigen in the external water phase and the vegetable oil in the internal oil phase are slowly released. Similarly, the tendencies of the APPs are in accordance with the levels of the proinflammatory cytokines IL-1β and TNF-α (Figure 1D), potentially because these proinflammatory cytokines serve as signals to hepatocytes to produce APPs that are then released into blood circulation [28]. The relationship between changes in the levels of APPs and proinflammatory cytokines provides information to assess the safety of vaccines for clinical use. In this study, the side effects of adjuvant and vaccines were trivial and lasted only a short time, inducing only a weak and transient local inflammatory reaction. However, several other cytokines, such as IFN-γ, IL-2 and IL-6, are still present at day 28 in experiment B. While these cytokines are necessary to induce immune responses against *Bb*, their long-term presence in the sera, where responses may be non-specific, may also indicate toxicity (autoimmunity). As such, more research is needed to further explore the possible reasons.

The vegetable origin oils, such as sesame oil, cottonseed oil, and castor oil, have been used in approved products [1]. Recently, the good tolerance of vegetable oil was also verified for a sunflower seed oil-containing adjuvant in chickens [34]. In conclusion, the above indicators demonstrate that the soybean oil-based adjuvant E515 and the E515-adjuvanted *Bb* vaccine had good safety profiles in rabbits. The toxicity parameters assessed in this study are limited. Next, the morphological and histological evaluation of organs, and hematological toxicity will be further assessed.

In our previous studies, E515 as an FMD vaccine adjuvant was shown to activate vigorous humoral and cellular responses [20,21]. Based on the good safety of the E515-*Bb* vaccine in rabbits, its immune effects were further demonstrated by evaluating antigen-specific humoral and cellular responses on days 7, 14, 21, and 28 post-boost immunization. The humoral immune response of IgG can provide protection against *Bb* infection [13,35]. The antibody results reveal that both adjuvant groups exhibited excellent humoral responses throughout the whole experiment, whereas the IgG and IgG isotype levels induced by the E515-*Bb* vaccine were superior to those induced by the Alum-*Bb* vaccine, and the antibody response peaked at 14 days post-boost (Figure 2A). Soluble CD4 and CD8 have been suggested to be released into the serum from activated CD4 and CD8 T cells [36,37]. Bacterial vaccines can reportedly enhance the activities of both CD4 and CD8 T cells and result in better protective immunity [38]. In this study, soluble CD4 and CD8 were produced at significantly higher levels in the E515-*Bb* vaccine group than in the other groups (Figure 2B), indicating that the E515-*Bb* vaccine induced a strong immune response at the cellular level.

The immune system is a network comprised of various specialized cell types that communicate via cytokines to perform specific types of defensive responses. Th1 cells mainly produce IFN-γ and IL-2, and Th2 cells are responsible for humoral immunity by producing IL-4 and IL-6 [39]. Thus, the increased levels of the IgG antibody in the E515-*Bb* group might have been related to the higher cytokine concentrations of IFN-γ, IL-2, IL-4 and IL-6 compared to those in the other groups (Figure 2C). Moreover, the IL-17 cytokine levels were significantly improved in the E515-*Bb* group compared with the Alum-*Bb* group except on day 28 (Figure 2C). IL-17 is produced by Th17 cells and plays an important role in defending against gram-negative bacterial infection [40]. This study indicates that the E515-adjuvanted *Bb* vaccine activates Th1/Th2/Th17 cell responses. Recently, a virus vaccine containing GS adjuvant was found to evoke balanced Th1/Th2/Th17 responses in a mouse model [41]; thus, the role of GS in the effects of the E515 adjuvant is worthy of further exploration.

A suitable vaccine should effectively protect animals from pathogen infection [42]. Then, we assessed the immunoprotective effect of the E515-*Bb* vaccine by monitoring the eventual death of the rabbits and the serum endotoxin and proinflammatory cytokine levels post *Bb* challenge. The E515-*Bb* vaccine enhanced the resistance of rabbits to infection by *Bb*, achieving a 100% rabbit survival rate (Figure 3A). During infection, increasing levels of endotoxin and proinflammatory cytokines induced by bacterial virulence factors gradually cause inflammation, which could result in septicemia and even animal death [43,44]. Our results indicate that E515 suppresses the serum endotoxin levels and decreases the levels of proinflammatory cytokines (Figure 3B,C), thereby inhibiting *Bb* infection and improving the survival rate of rabbits. Overall, the E515-*Bb* vaccine exerts an excellent immune effect on rabbits to protect against *Bb* infection.

Adjuvants play significant roles in vaccine formulations to provide long-term protection [44,45,46,47]. An ideal adjuvant should promote the early induction of a humoral immune response by a vaccine that is long lasting and strengthens vaccine-mediated protection against pathogen infection [48]. We found that the E515-adjuvanted *Bb* vaccine induces long-lasting *Bb*-specific antibodies for up to 6 months, and the IgG levels peak at the first month post-vaccination; in the Alum-*Bb* vaccine group, the levels progressively peaked at 2 months after immunization. This result indicates that the *Bb* vaccine formulated with the E515 adjuvant induced the production of the antibody response earlier than the Alum adjuvant (Figure 4A). The rapid, enhanced, and long-lasting immune response stimulated by the E515-adjuvanted *Bb* vaccine might be related to the gradual release of *Bb* antigen and the prolonged exposure of antigens that are beneficial to immune cells, and similar to the immunological effect of Montanide series of oil adjuvants in inactivated vaccines [48]. Furthermore, significantly reduced *Bb* counts in the lung and trachea and normal lung tissue structures were observed in the group receiving the *Bb* vaccine adjuvanted with E515 compared to those rabbits receiving the nonadjuvanted vaccine (Figure 4B,C), which suggests that vaccination with E515-*Bb* decreased the inflammatory responses in the lung and slowed the pathological damage in the tissue. This result may have been correlated with a successful long-lasting humoral immune response stimulated by the E515 adjuvant vaccine [21], and similar results were reported for a conjugate bacterial vaccine [44]. Moreover, Th1/Th17 cellular responses are required for long-lasting protective immunity against *Bb* and other bacterial pathogens [35,49]. Thus, the Th1/Th17 cellular responses improved by the E515-adjuvanted vaccine may offer efficacious and durable immune responses against bacterial pathogens.

RNA-seq-based transcriptomics allows the precise determination of transcript levels and has been used to elucidate the roles of adjuvants in various vaccine immunizations [50,51,52]. Moreover, proteomic technology provides information regarding changes in protein expression and posttranslational regulation. The 4D label-free quantitative proteomic technique, which has a very high sensitivity and accurate quantification capability, can detect more low-abundance proteins than normal proteomic methods [53,54,55]. In this study, RNA-seq and 4D label-free proteomics were adopted to investigate the role of the E515 adjuvant in the E515-*Bb* vaccine in defense against *Bb* challenge. Compared to the *Bb* antigen group, the E515-*Bb* group exhibited more DEGs and DEPs (Figures S2 and S6). The GO terms positive regulation of T cell differentiation, cellular response to cytokines (TGF, IL-4), regulation of leukocyte and lymphocyte activation, activation of immune response and apoptotic cell clearance were obviously identified in the E515-*Bb* group when compared with the *Bb* or Alum-*Bb* group (Tables S2 and S3), which further confirmed the enhanced cellular responses induced by the E515 adjuvant compared with Alum.

To gain insight into the signaling pathways involved in the response to the E515-adjuvanted *Bb* vaccine, KEGG enrichment analysis was conducted [21]. The pathways identified in the E515-*Bb* group by RNA-seq were mainly involved in DNA replication, protein processing and the cell cycle, and these pathways were all downregulated when compared to those in the other two groups (Figure 5). This suggests that the E515-*Bb* vaccine downregulates genetic information processing and cellular processes to protect rabbits from *Bb* invasion. In addition, ribosome and ECM−receptor interaction pathways were significantly enriched in the E515-*Bb* vaccine group compared with the *Bb* group at the protein level (Figure 6A). Our previous study showed that DEPs (L23, L24, L37, S24, and S25) were significantly upregulated in the ribosome pathway in the *Bb*-challenged group compared to the control group [23]. After *Bb* challenge, the ribosome pathway was significantly downregulated in E515-*Bb* vs. *Bb*, demonstrating that E515 might interfere with ribosome protein synthesis to inhibit bacterial replication in splenocytes. In the E515-*Bb* vs. *Bb* comparison, the ECM−receptor interaction pathway was obviously upregulated. The ECM−receptor interaction pathway plays a critical role in the maintenance of tissue, organ and cell morphogenesis, structure, and function [56]. After challenge, *Bb* produces endotoxins that are damaging to rabbit tissues. Moreover, various cells migrate into the susceptible tissues, leading to destruction of the lung and trachea. Therefore, upregulating DEPs in this pathway could be an important mechanism to reduce tissue damage caused by *Bb* infection. These results may be beneficial for understanding the lower serum levels of endotoxin, the reduced *Bb* burden in the lung and the fewer lung lesions in rabbits injected with the E515-*Bb* vaccine compared with the *Bb* antigen (Figure 4B,C). In addition, the B cell receptor signaling pathway was significantly upregulated in E515-*Bb* vs. Alum-*Bb* (Figure 6C). This finding is consistent with the results of phenotypic assays that showed higher levels of *Bb*-specific antibody production induced by the E515-*Bb* vaccine.

In our previous studies, RNA-seq-based transcriptomic and tandem mass tags (TMT) proteomic analyses were used to investigate the mechanisms underlying the adjuvant in mouse models and Hu sheep, respectively [20,21]. However, generally single omics technologies cannot always comprehensively reveal the potential molecular mechanisms. Integrating multi-omics analyses is critical to elucidating gene and protein regulation networks from a global viewpoint. Transcriptome and proteome analyses in combination has been widely applied in many research fields, such as human and animal physiology and pathology research [57,58,59]. To the best of our knowledge, no studies have reported the use of multi-omics to analyze the effects of vegetable oil adjuvants. In this study, a comprehensive multi-omics approach was used to elucidate the potential mechanism by which the E515 adjuvant vaccine protects against *Bb* infection in rabbits. Among the DEGs/DEPs identified in the E515-*Bb* vs. *Bb* comparison, 52 out of 64 cor-DEGs-DEPs showed the same expression trend, and 12 showed the opposite trend. However, only two genes were co-expressed in the two adjuvant groups, which showed that they had obviously different adjuvant effects on the *Bb* vaccine (Figure 7).

DEGs in the transcriptome may not be expressed in the proteome and vice versa. Therefore, DEGs and DEPs changed at both the transcriptome and proteome levels were selected for subsequent immune-related KEGG pathway enrichment analysis. The complement and coagulation cascades are vital mediators of the innate immune response to pathogens. The main consequences of complement activation are the opsonization of pathogens, the recruitment of inflammatory and immunocompetent cells, and the direct killing of microorganisms, such as bacteria, which is a primary line of defense against infection [60,61]. A study of bovine mammary glands naturally infected with *Staphylococcus aureus* showed that the protein expression of complement component 3 (C3) and the complement and coagulation cascades pathway were significantly downregulated in the mastitis group [56]. CAMs play a critical role in the immune response, which includes antigen recognition, costimulation, and cellular adhesion (https://www.genome.jp/entry/map04514). Our results indicate that in the E515-*Bb* vs. *Bb* comparison, complement C1q B (G1T8T5) expression was significantly upregulated, and the pathways of complement and coagulation cascades and CAMs were significantly upregulated in both the transcriptome and proteome analyses (Figure 8 and Figure 9A). The P53 pathway can be activated by stress signals, such as DNA damage and oxidative stress and results in cell cycle arrest, cellular senescence or apoptosis (https://www.genome.jp/entry/map04115). Notably, the downregulated pathways herein, including the base excision repair, DNA replication, p53 signaling, protein processing in the endoplasmic reticulum, and cell cycle pathways, were significantly enriched in the E515-*Bb* vs. *Bb* comparison (Figure 9B). Therefore, the good immune-protective effect observed in the E515-*Bb* group was potentially due to the E515 adjuvant preventing the disruption of DNA replication and cell division and downregulating the cell cycle arrest program, cellular senescence, or apoptosis [62], and the upregulated proteins in the complement and coagulation cascades and CAM pathways facilitate the direct killing of *Bb* to better protect rabbits from *Bb* infection.

## 5. Conclusions

In summary, the *Bb* vaccine adjuvanted with E515 exhibited good safety and effectively induced immune responses against *Bb* infection in rabbits. The s.c. administration of double-doses of E515 alone or E515-*Bb* was well tolerated in rabbits. Furthermore, rabbits immunized with the E515-*Bb* vaccine exhibited significantly higher, earlier and long-lasting antibody responses and Th1/Th2/Th17 cell responses than those immunized with the Alum-*Bb* vaccine or *Bb* antigen. Moreover, the E515-adjuvanted *Bb* vaccine provided effective protection against *Bb* infection in rabbits. Transcriptome and proteome analyses in combination highlighted that the pathways associated with the complement and coagulation cascades, CAMs and the P53 pathway were obviously enriched in the E515-*Bb* vaccine group, which could facilitate our understanding of the adjuvant mechanism of E515. Overall, the vegetable-derived E515-adjuvanted *Bb* vaccine should be considered a promising candidate vaccine for preventing *Bb* infection.

## Data Availability

Data are available from the authors upon reasonable request.

## Supplementary Materials

The following supporting information can be downloaded at: https://www.mdpi.com/article/10.3390/pharmaceutics14071434/s1. Figure S1: Experimental design. (A) Safety estimation of the adjuvanted *Bb* vaccine. Rabbits (*n* = 9/group) were immunized subcutaneously (s.c.) on days 0 and 14. Temperature and/or weight changes were monitored. Serum samples were collected for acute phase proteins and cytokines detection at different time points. (B) Immune effects of the E515-*Bb* vaccine. Rabbits (*n* = 9/group) were immunized (s.c.) on days 0 and 14. Serum samples were collected to detect antibody responses and cellular responses. On day 28, the rabbits were challenged with live *Bb*. The animal survival rate was monitored, serum samples were collected for endotoxin and/or cytokines detection, spleen samples of rabbits (*n* = 3/group) were collected for proteomic and transcriptomic analyses. (C) Long-term protection response of E515-*Bb* vaccine. Rabbits (*n* = 9/group) were immunized (s.c.) on day 0. Serum samples were collected to detect *Bb*-specific IgG at different time points. On day 240, rabbits were challenged with live *Bb*, seven days later lung and trachea were collected for bacterial burdens quantified and HE staining. Figure S2: The numbers of differentially expressed genes (DEGs). Rabbits (*n* = 9/group) were s.c. injected twice at a 2-week interval with inactivated *Bb* antigen (1.2 × 10^10^ CFU/mL), or inactivated *Bb* antigen adjuvanted with E515 or Alum. Rabbits were challenged 4 weeks after boosting by ear vein injection of live *Bb* (2.4 × 10^10^ CFU/rabbit). Spleens (*n* = 3/group) were harvested 10 days after infection and used for transcriptomic sequencing. The histogram shows the numbers of up and downregulated DEGs in two comparisons. Figure S3: Heat map of hierarchical clustering of DEGs in two comparisons. The red color indicates high expression, the blue color indicates low expression. Figure S4: Identification and quantitation of the proteome. “Total spectrum” is the total number of the secondary mass spectra, and “Matched spectrum” is the number of the secondary mass spectra after quality control. “Peptide” is the number of the identified peptides, “Unique peptide” is the number of the identified peptides which belong only to a group of proteins, “Identified protein” is the number of identified proteins, “Quantifiable proteins” is the number of quantifiable proteins. Figure S5: Box-plot showing the repeatability within each group. Figure S6: The numbers of differentially expressed proteins (DEPs). The histogram shows the numbers of up- and downregulated DEPs in two comparisons. Figure S7: KEGG pathway enrichment-based clustering analysis of DEGs and DEPs in Alum-*Bb* vs. *Bb*. Figure S8: KEGG pathway enrichment-based clustering analysis of DEGs and DEPs in E515-*Bb* vs. Alum-*Bb*. Figure S9: Validation of DEGs/DEPs by RT-qPCR/PRM. Fold change of relative mRNA/protein expression levels by RNA-seq and RT-qPCR (A), or 4D label-free and PRM (B). Table S1: Sequences of primers for quantitative RT-qPCR. Table S2: Transcriptome GO enrichment. Table S3: Proteome GO enrichment. Table S4: Proteome & Transcriptome KEGG pathway enrichment.
