# Supplementary material for: Safety and Efficacy of the Bordetella bronchiseptica Vaccine Combined with a Vegetable Oil Adjuvant and Multi-Omics Analysis of Its Potential Role in the Protective Response of Rabbits
Source: Pharmaceutics. 2022 Jul 8;14(7):1434. doi: 10.3390/pharmaceutics14071434 (PMC9317422; doi:10.3390/pharmaceutics14071434)
Supplement: Supplementary file 1 [file pharmaceutics-14-01434-s001.zip › supplementary figures.pdf]

Supplementary Data

Safety and Efficacy of the *Bordetella bronchiseptica* Vaccine Combined with a Vegetable Oil Adjuvant and Multiomics Analysis of Its Potential Role in the Protective Response of Rabbits

Xuemei Cui, Xiangfei Xu, Pan Huang, Guolian Bao, Yan Liu

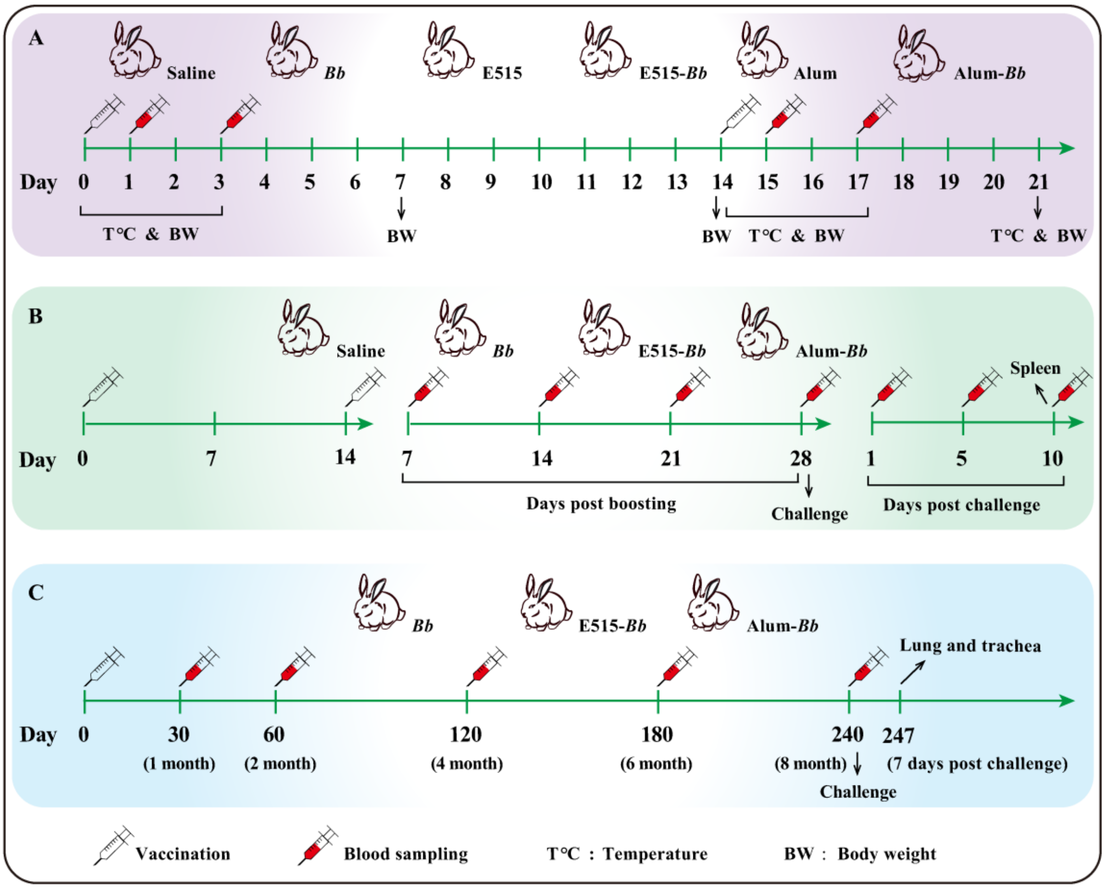

Figure S1. Experimental design. (**A**) Safety estimation of the adjuvanted *Bb* vaccine. Rabbits (n = 9/group) were immunized subcutaneously (s.c.) on days 0 and 14. Temperature and/or weight changes were monitored. Serum samples were collected for acute phase proteins and cytokines detection at different time points. (**B**) Immune effects of the E515-*Bb* vaccine. Rabbits (n = 9/group) were immunized (s.c.) on days 0 and 14. Serum samples were collected to detect antibody responses and cellular responses. On day 28, the rabbits were challenged with live *Bb*. The animal survival rate was monitored, serum samples were collected for endotoxin and/or cytokines detection, spleen samples of rabbits (n = 3/group) were collected for proteomic and transcriptomic analyses. (**C**) Long-term protection response of E515-*Bb* vaccine. Rabbits (n = 9/group) were immunized (s.c.)

on day 0. Serum samples were collected to detect *Bb*-specific IgG at different time points. On day 240, rabbits were challenged with live *Bb*, seven days later lung and trachea were collected for bacterial burdens quantified and HE staining.

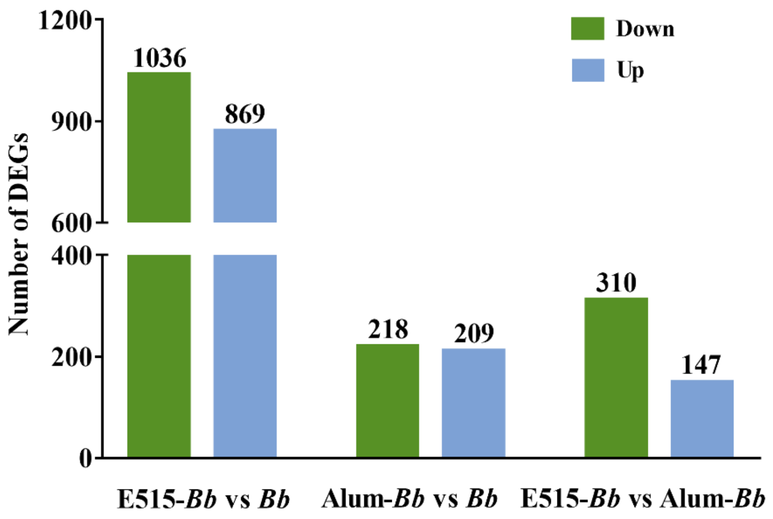

Figure S2. The numbers of differentially expressed genes (DEGs). Rabbits (n = 9/group) were s.c. injected twice at a 2-week interval with inactivated *Bb* antigen (1.2×10^{10} CFU/mL), or inactivated *Bb* antigen adjuvanted with E515 or Alum. Rabbits were challenged 4 weeks after boosting by ear vein injection of live *Bb* (2.4×10^{10} CFU/rabbit). Spleens (n = 3/group) were harvested 10 days after infection and used for transcriptomic sequencing. The histogram shows the numbers of up and downregulated DEGs in two comparisons.

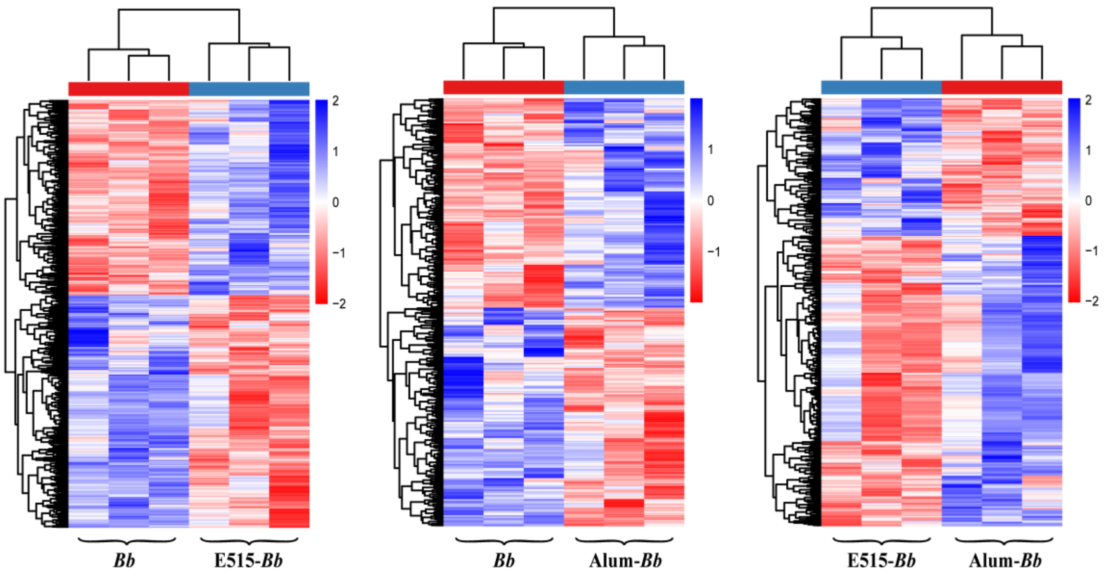

Figure S3. Heat map of hierarchical clustering of DEGs in two comparisons. The red color indicates high expression, the blue color indicates low expression.

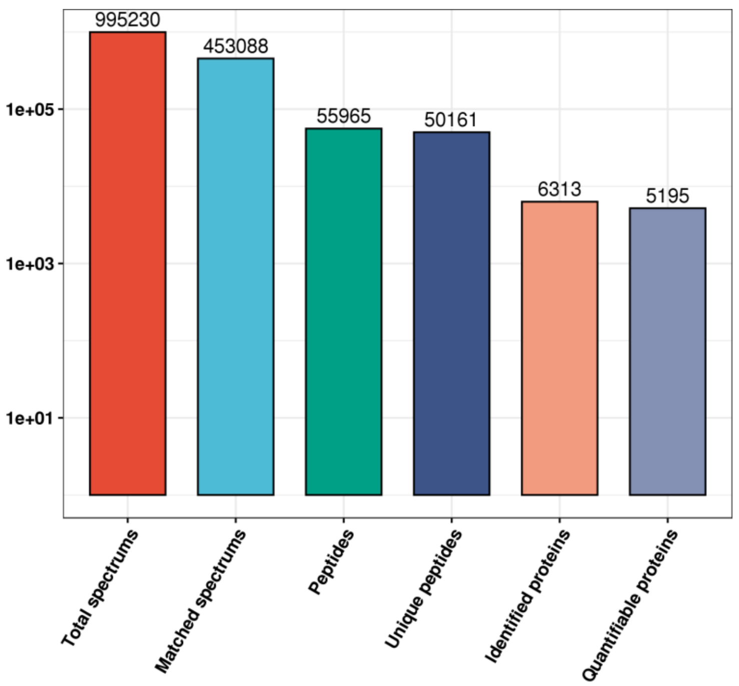

Figure S4. Identification and quantitation of the proteome. “Total spectrum” is the total number of the secondary mass spectra, and “Matched spectrum” is the number of the secondary mass spectra after quality control. “Peptide” is the number of the identified peptides, “Unique peptide” is the number of the identified peptides which belong only to a group of proteins, “Identified protein” is the number of identified proteins, “Quantifiable proteins” is the number of quantifiable proteins.

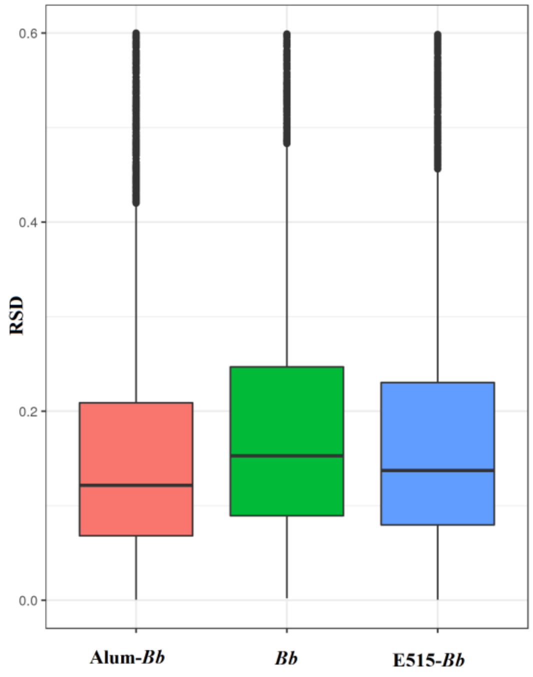

Figure S5. Box-plot showing the repeatability within each group.

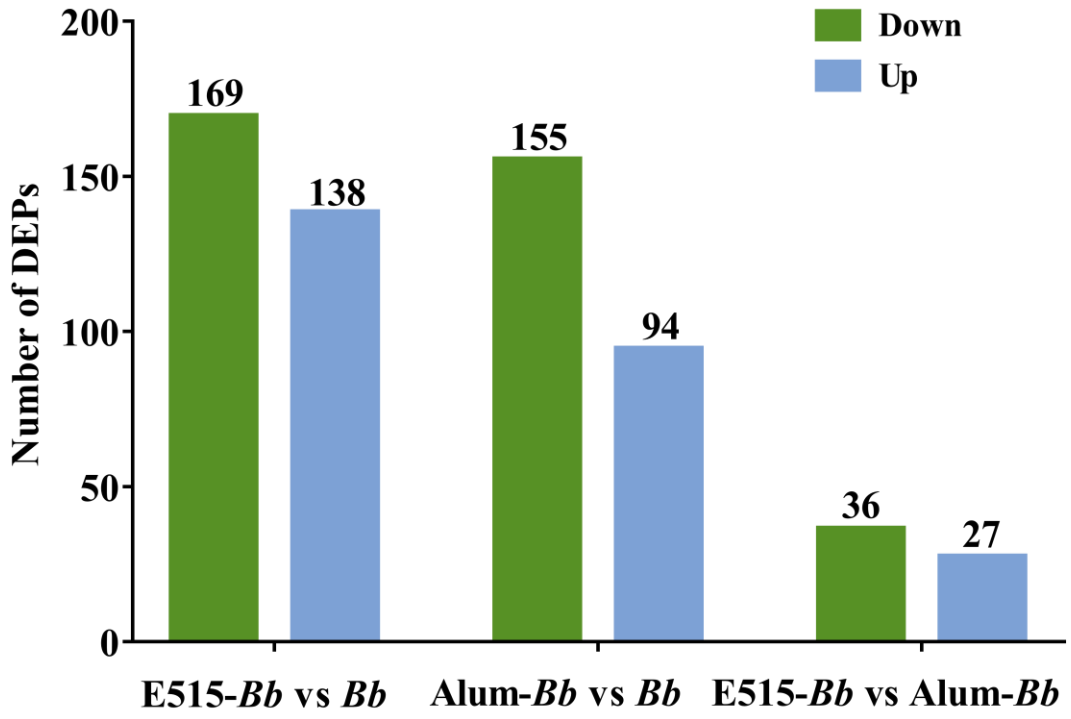

Figure S6. The numbers of differentially expressed proteins (DEPs). The histogram shows the numbers of up- and downregulated DEPs in two comparisons.

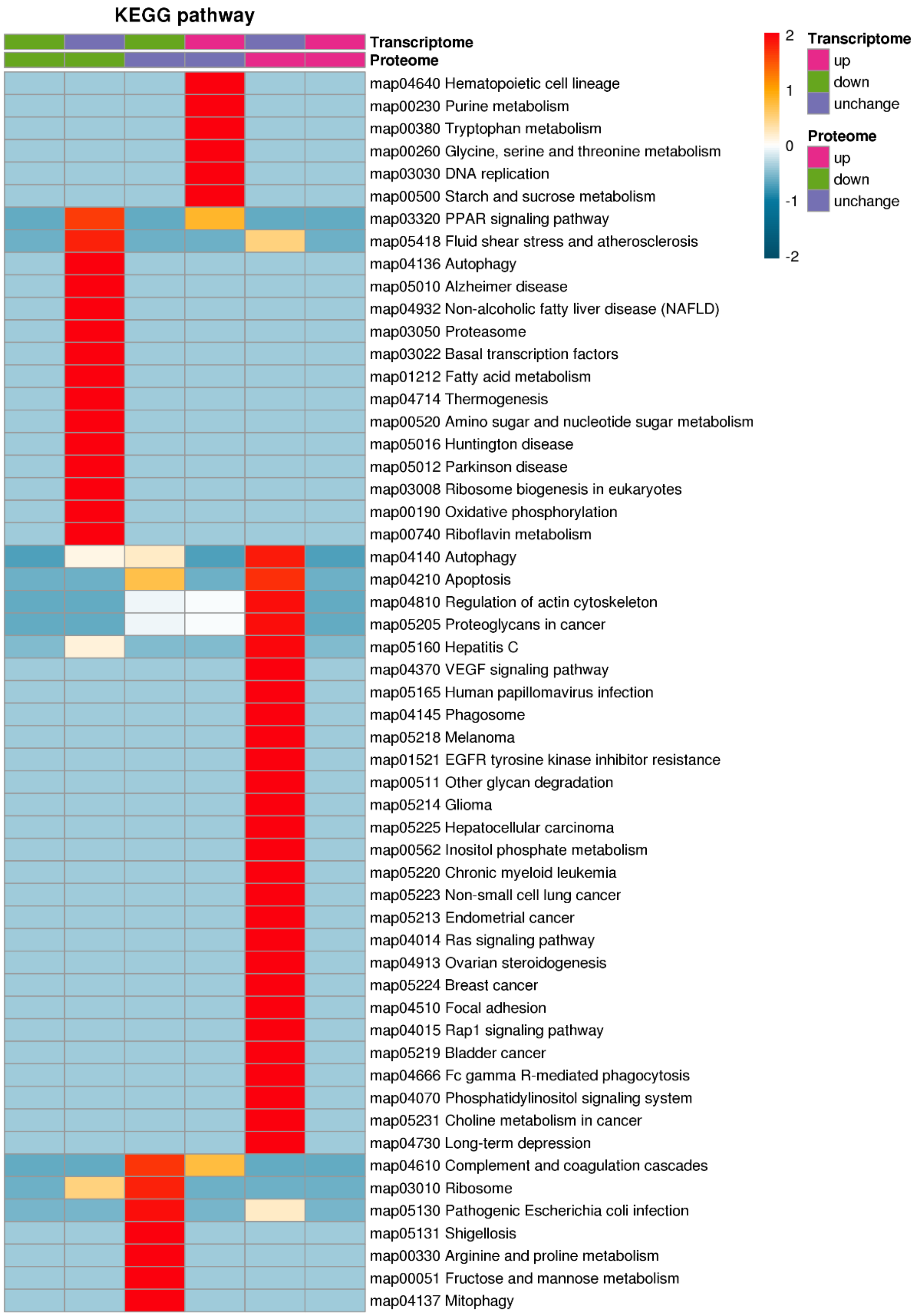

Figure S7. KEGG pathway enrichment-based clustering analysis of DEGs and DEPs in Alum-*Bb* vs. *Bb*.

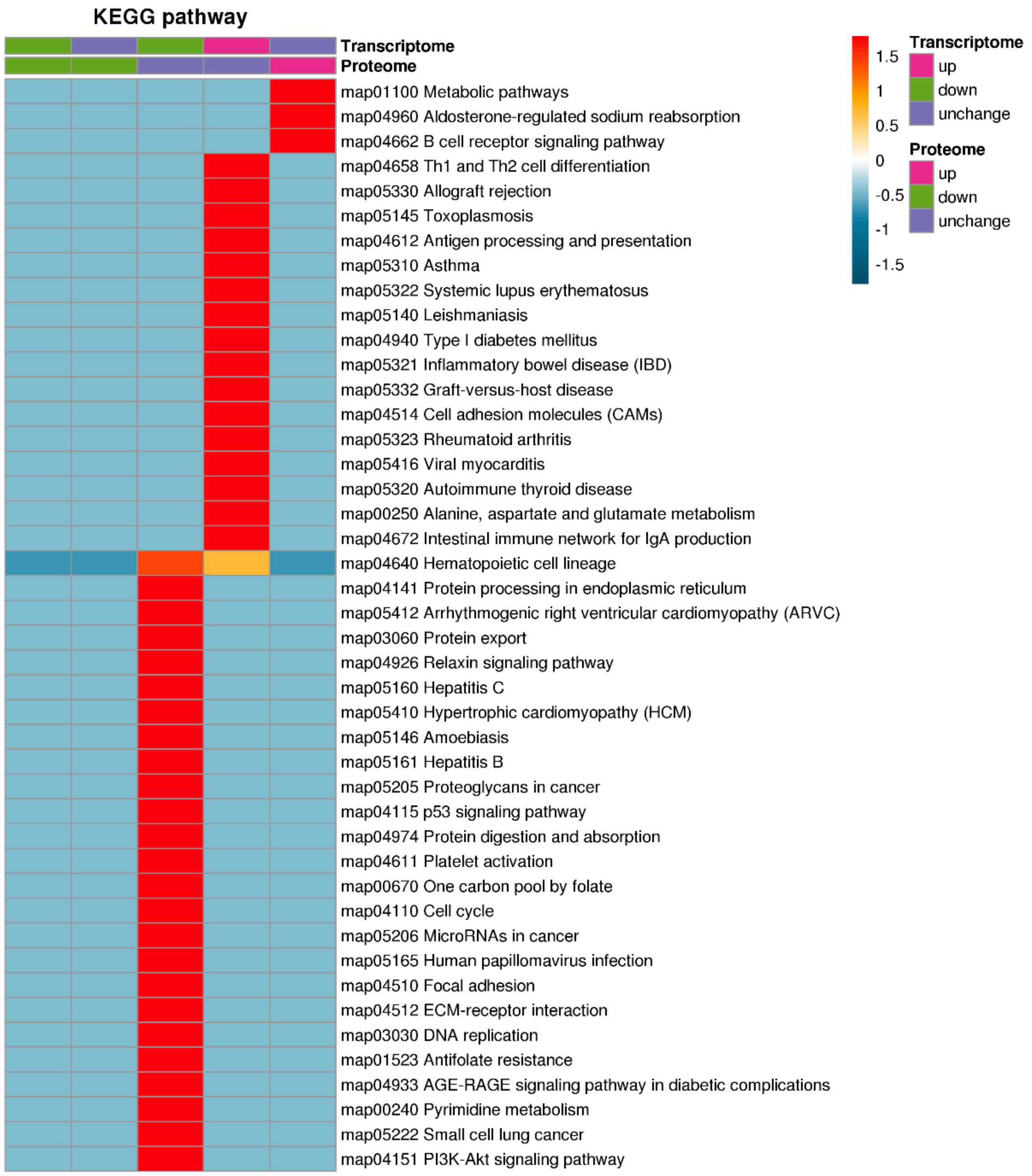

Figure S8. KEGG pathway enrichment-based clustering analysis of DEGs and DEPs in E515-*Bb* vs. Alum-*Bb*.

A

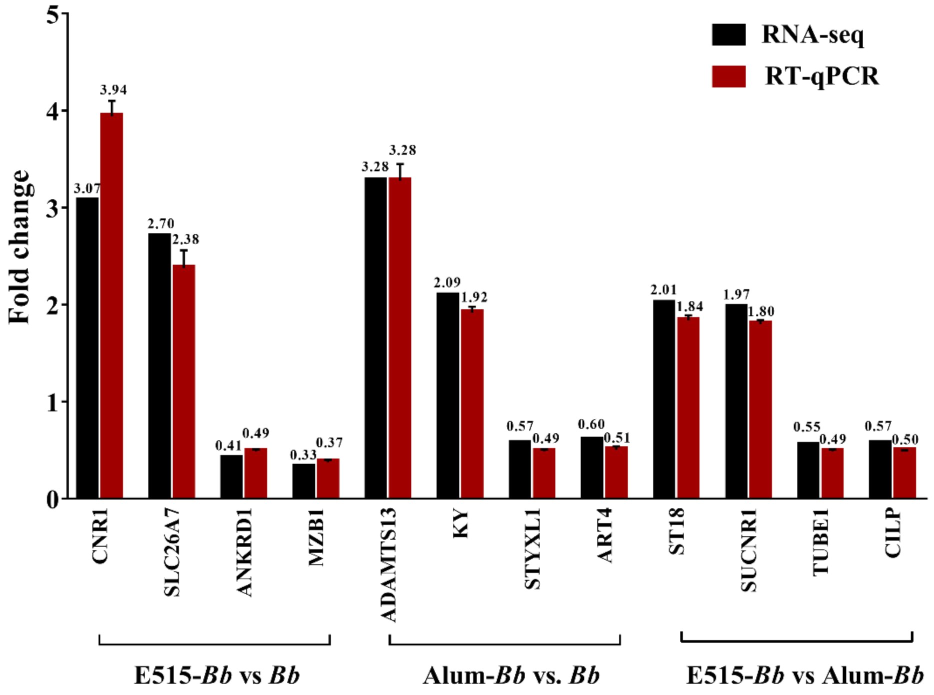

B

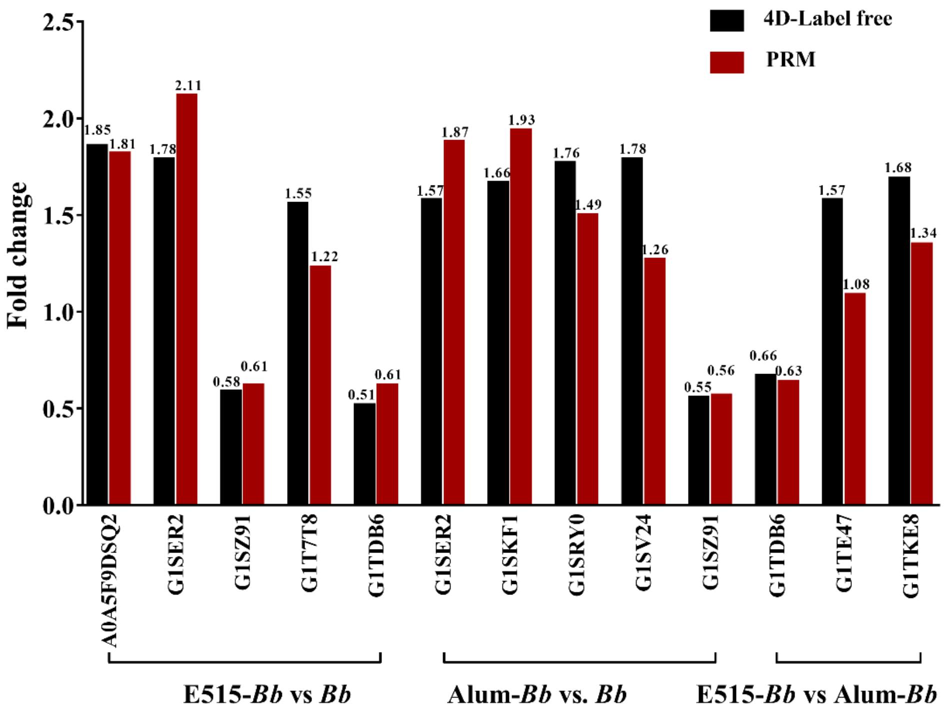

Figure S9. Validation of DEGs/DEPs by RT-qPCR/PRM. Fold change of relative mRNA/protein expression levels by RNA-seq and RT-qPCR (**A**), or 4D label-free and PRM (**B**).
